# Genetically engineered human muscle transplant enhances murine host neovascularization and myogenesis

**DOI:** 10.1038/s42003-018-0161-0

**Published:** 2018-10-04

**Authors:** Luba Perry, Shira Landau, Moshe Y. Flugelman, Shulamit Levenberg

**Affiliations:** 1 Biomedical Engineering Department, Technion-Israel Institute of Technology, Haifa, 32000 Israel; 2 Inter-departmental Program in Biotechnology, Technion-Israel Institute of Technology, Haifa, 32000 Israel; 3 Department of Cardiovascular Medicine, Lady Davis Carmel Medical Center, Haifa, 3436212 Israel

## Abstract

Engineered tissues are a promising tool for addressing the growing need for tissues and organs in surgical reconstructions. Prevascularization of implanted tissues is expected to enhance survival prospects post transplantation and minimize deficiencies and/or hypoxia deeper in the tissue. Here, we fabricate a three-dimensional, prevascularized engineered muscle containing human myoblasts, genetically modified endothelial cells secreting angiopoietin 1 (ANGPT1) and genetically modified smooth muscle cells secreting vascular endothelial growth factor (VEGF). The genetically engineered human muscle shows enhanced host neovascularization and myogenesis following transplantation into a mouse host, compared to the non-secreting control. The vascular, genetically modified cells have been cleared for clinical trials and can be used to construct autologous vascularized tissues. Therefore, the described genetically engineered vascularized muscle has the potential to be fully translated to the clinical setting to overcome autologous tissue shortage and to accelerate host neovascularization and integration of engineered grafts following transplantation.

## Abstract (web summary)

Luba Perry et al. report transplantation of engineered prevascularized human muscle into mice to repair an abdominal muscle defect. They show that genetically engineering smooth muscle cells to secrete VEGF and endothelial cells to secrete ANGPT1 significantly improves host neovascularization and myogenesis.

## Introduction

Muscle atrophy can result from severe traumatic events, such as deep burns and cancer, and requires surgical reconstruction. To date, use of autologous flaps is the gold standard treatment in reconstructive surgery, but its use is limited by low anatomic availability and donor site morbidity^1–4^. Engineered tissue grafts present a clinically relevant alternative^5–7^, with current research continuously improving their quality and effectiveness. Since vascularization is essential for maintenance of implant viability following transplantation^8–12^, efforts have been invested in design of prevascularized engineered tissue. One such approach employs multicellular culturing of endothelial cells with mural and tissue-specific cells, to induce self-assembly of vessel networks^8,10,13^. In order to create long-lasting, stable blood vessels, endothelial cells must be co-cultured with mural cells^14–17^. Moreover, vast neovascularization by the host and faster host vessel maturation was observed upon transplantation of endothelial cell–mural cell co-cultures, as compared to endothelial cell or mural cell mono-culture graft transplantation^18^. We have previously reported on construction of a three-dimensional (3D) muscle graft from human adult and differentiated cells, which then seamlessly integrated with native muscle tissue upon transplantation^12^. In parallel, intra-arterial injection of adult venous endothelial cells, transduced to express angiopoietin 1 (ANGPT1), with adult venous smooth muscle cells (SMC), transduced to express vascular endothelial growth factor (VEGF_165_), proximal to an occluded artery in critical limb ischemia patients stimulated collateral expansion^19,20^. In the current report, we fabricated a 3D prevascularized engineered muscle containing human myoblasts. The addition of myoblasts to the endothelial cell–mural cell co-culture has been shown to increase the mechanical strength of the transplanted tissue^21^. We aimed to construct an engineered muscle graft, composed entirely of human cells, with a high density of functional blood vessels to improve oxygen and nutrient delivery to the graft. We hypothesized that seeding a multicellular culture of human ANGPT1-expressing endothelial cells (endothelial cell^ANGPT1^) with human VEGF-expressing SMCs (SMC^VEGF^) and human myoblasts on 3D polymer scaffolds would accelerate both in vitro vessel-like network formation and post-implantation neovascularization of the transplant. To this end, the maturity and complexity of vessel-like structures formed in scaffolds bearing all three cell types were assessed. In addition, host neovascularization was monitored, via an abdominal imaging window (AIW), and characterized for up to 14 days after transplantation of such scaffolds into an abdominal wall defect of mice. Immunodeficient nude mice were used to study human graft integration while avoiding transplant rejection. Endothelial cell^ANGPT1^ and SMC^VEGF^ demonstrated greater vasculogenic potential than naive cells. The vascular, genetically engineered cells used to fabricate the vascularized engineered muscle can be easily isolated from short human vein segments and used to construct an autologous vascular tissue that promotes its neovascularization and integration following transplantation. Moreover, these cells have been cleared by the Food and Drug Administration for clinical trials. Therefore, these findings may have profound clinical implications in the construction of autologous, transplantable grafts, and in the prospects and rate of their integration and vascularization following transplantation.

## Results

### Genetically modified vascular cells secrete ANGPT1 and VEGF

**Fig. 1:**
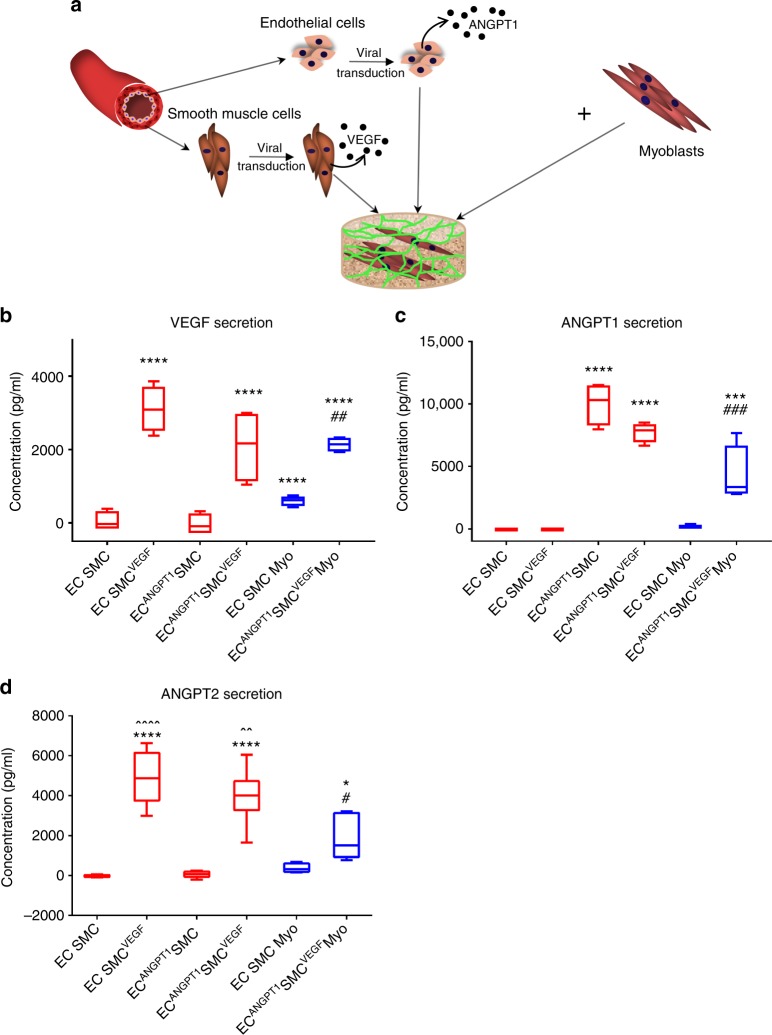
Vascular cell transduction and multicellular culturing strategy. **a** A schematic presentation of the different multicellular cultures examined: co-culture of naive or ANGPT1-secreting endothelial cells, with naive or VEGF-secreting SMCs, were cultured to generate 3D vascular networks. Tri-cultures of naive or ANGPT1-secreting endothelial cells, with naive or VEGF-secreting SMC and human myoblasts, were cultured to generate 3D vascular networks within skeletal muscle constructs. **b** Secreted VEGF concentrations, as quantified by ELISA, 4 days after co- and tri-culture seeding on PLLA/PLGA scaffolds. EC endothelial cells, SMC smooth muscle cells, Myo myoblasts. Data are expressed as box-and-whisker plots, where the central lines denote medians, edges represent upper and lower quartiles and whiskers show minimum and maximum values. Data were analyzed by one-way ANOVA, followed by Holm–Sidak’s multiple comparison test, *n* = 4 (^##^*p* = 0.0045 versus EC-SMC-Myo tri-culture, ****p < 0.0001 versus EC-SMC co-culture). **c** Secreted ANGPT1 concentrations, as quantified by ELISA, 4 days after co- and tri-culture seeding on PLLA/PLGA scaffolds. Data are expressed as box-and-whisker plots, where the central lines denote medians, edges represent upper and lower quartiles and whiskers show minimum and maximum values. Data were analyzed by one-way ANOVA, followed by Holm–Sidak’s multiple comparison test, *n* = 4 (^###^*p* = 0.0009 versus EC-SMC-Myo tri-culture, ***p = 0.0007 versus EC-SMC co-culture, ****p < 0.0001 versus EC-SMC co-culture). **d** Secreted ANGPT2 concentrations, as quantified by ELISA, 4 days after co- and tri-culture seeding on PLLA/PLGA scaffolds. Data are expressed as box-and-whisker plots, where the central lines denote medians, edges represent upper and lower quartiles and whiskers show minimum and maximum values. Data were analyzed by one-way ANOVA, followed by Holm–Sidak’s multiple comparison test, *n* = 6 (^#^*p* = 0.0471 versus EC-SMC-Myo tri-culture, *p = 0.0104 versus EC-SMC co-culture, ****p < 0.0001 versus EC-SMC co-culture, ^^*p* = 0.0033 versus EC^ANGPT1^-SMC^VEGF^-Myo tri-culture, ^^^^*p* < 0.0001 versus EC^ANGPT1^-SMC^VEGF^-Myo tri-culture)

To confirm that the genetically modified vascular cells overexpress ANGPT1 and VEGF, adult venous endothelial cells and SMCs, overexpressing ANGT1 and VEGF, respectively, were cultured in six different multicellular co- and tri-culture combinations (Fig. 1a). Enzyme-linked immunosorbent assay (ELISA) of culture supernatants established that VEGF and ANGPT1 were barely secreted by naive co-cultures (Fig. 1b, c). Naive tri-cultures secreted high levels of VEGF and very low levels of ANGPT1 (Fig. 1b, c). Co-cultures and tri-culture containing SMC^VEGF^ contained more VEGF compared to those including SMCs (Fig. 1b). Similarly, ANGPT1 concentrations were higher in endothelial cell^ANGPT1^-containing co-cultures and tri-cultures compared to those including endothelial cells (Fig. 1c). Since VEGF secretion is known to upregulate angiopoietin 2 (ANGPT2) expression^22^, we measured its levels in the above-mentioned culture supernatants (Fig. 1d). ANGPT2 levels were very low in both naive and endothelial cell^ANGPT1^ co-cultures compared to cultures with SMC^VEGF^ cells, and its levels were in correlation with the levels of VEGF (Fig. 1d).

### ANGPT1 promotes elongated vessel-like network formation

**Fig. 2:**
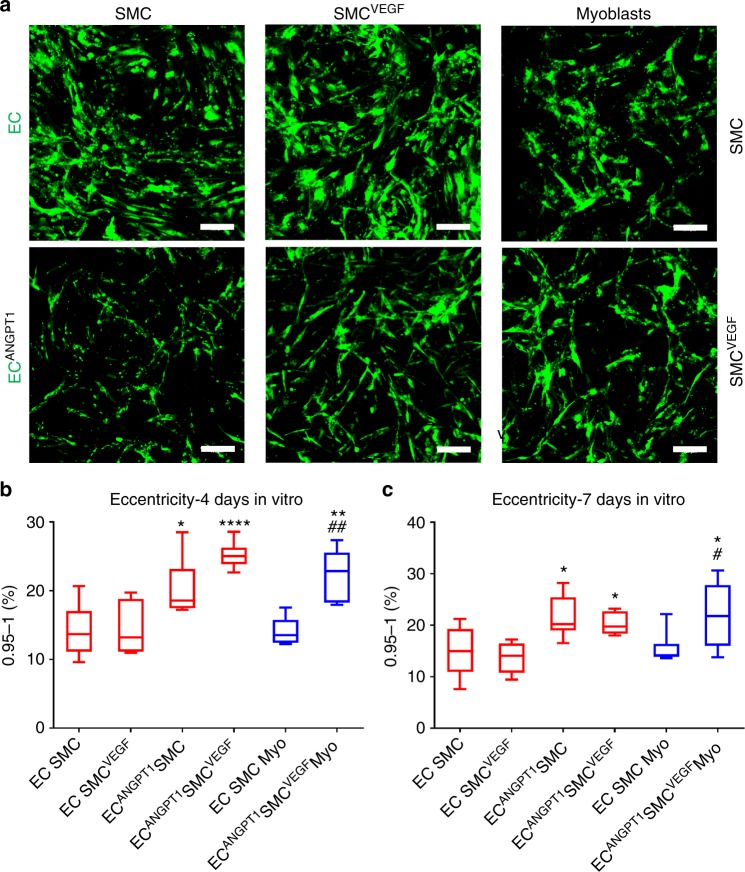
In vitro vessel-like network elongation. **a** Representative confocal images of scaffolds embedded with different cell combinations, imaged 4 days post seeding. EC endothelial cells, SMC smooth muscle cells. Endothelial cell-ZsGreen (naive or ANGPT1-secreting) are seen in green; scale bar = 100 µm. **b** Percentage of elongated elements with an eccentricity score of 0.95–1, 4 days post seeding. Myo myoblasts. Data are expressed as box-and-whisker plots, where the central lines denote medians, edges represent upper and lower quartiles and whiskers show minimum and maximum values. Data were analyzed by one-way ANOVA, followed by Holm–Sidak’s multiple comparison test, *n* = 6 (*p = 0.0336 versus EC-SMC co-culture, ^##^*p* = 0.0026 versus EC-SMC-Myo tri-culture, **p = 0.0027 versus EC-SMC co-culture, ****p < 0.0001 versus EC-SMC co-culture). **c** Percentage of elongated elements with an eccentricity score of 0.95–1, 7 days post seeding. Data are expressed as box-and-whisker plots, where the central lines denote medians, edges represent upper and lower quartiles and whiskers show minimum and maximum values. Data were analyzed by one-way ANOVA, followed by Holm–Sidak’s multiple comparison test, *n* = 6 (*p = 0.049 for EC^ANGPT1^-SMC versus EC-SMC co-culture, *p = 0.0316 for EC^ANGPT1^-SMCs versus EC-SMC^VEGF^ co-culture, *p = 0.0477 for EC^ANGPT1^-SMC^VEGF^-Myo tri-culture versus EC-SMC co-culture, ^#^*p* = 0.0475 versus EC-SMC-Myo tri-culture)

To evaluate the elongation of the vessel-like structures formed by various cell combinations in vitro, eccentricity measurements were performed on days 4 and 7 post seeding (Fig. 2 and Supplementary Fig. 1). Figure 2 displays the percentage of elements with eccentricity values of 0.95–1, indicative of elongated vessel structures, for all six tested cell combinations. Cultures with ANGPT1-expressing cells demonstrated higher percentage of elongated elements on both assessment days, compared to co- and tri-cultures containing naïve endothelial cells (Fig. 2b, c).

**Fig. 3:**
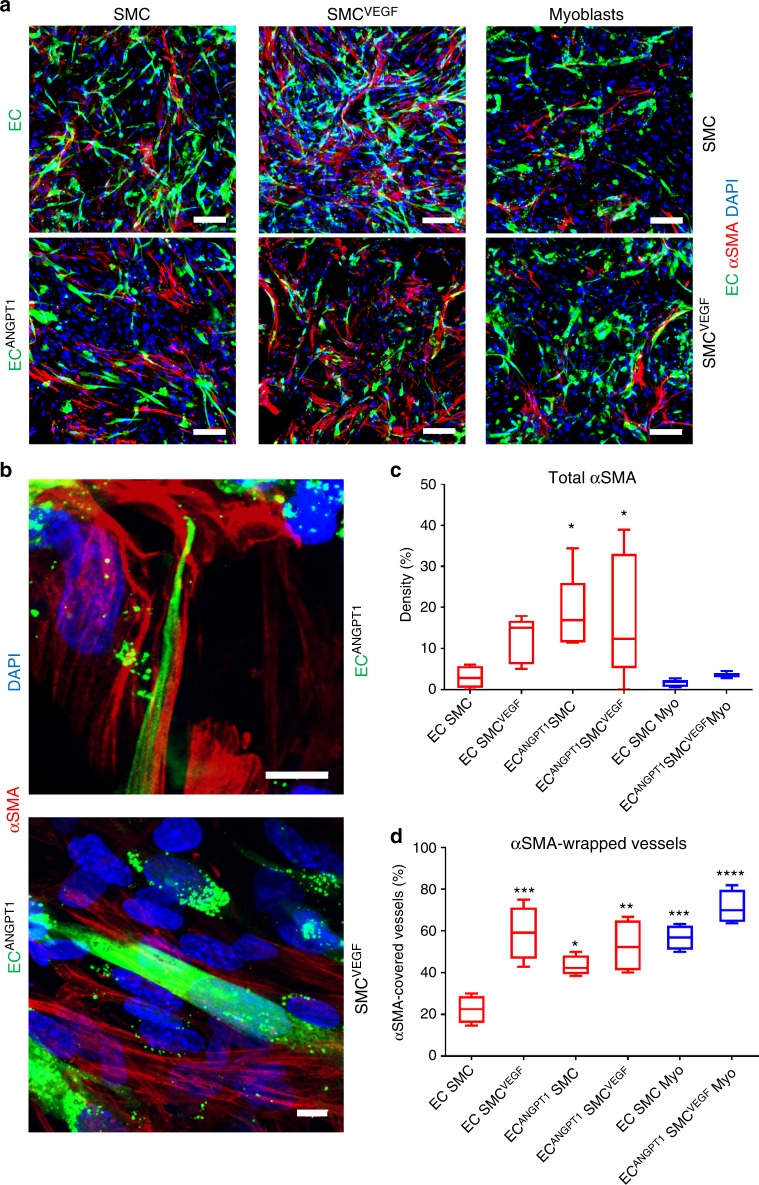
In vitro vessel-like network stability. **a** Representative confocal images of whole-mount immunofluorescent scaffolds populated with co- or tri-cultures. EC endothelial cells, SMC smooth muscle cells. Endothelial cell -ZsGreen are stained in green, αSMA-positive cells are stained in red and nuclei are stained in blue; scale bar = 100 µm. **b** Representative confocal images of whole-mount immunofluorescent scaffolds populated with endothelial cell^ANGPT1^ and SMCs^VEGF^. Endothelial cell-ZsGreen are stained in green, αSMA-positive cells are stained in red and nuclei are stained in blue; scale bar = 10 µm. **c** Total αSMA expression density. Myo myoblasts. Data are expressed as box-and-whisker plots, where the central lines denote medians, edges represent upper and lower quartiles and whiskers show minimum and maximum values. Data were analyzed by one-way ANOVA, followed by Holm–Sidak’s multiple comparison test, *n* = 5 (*p = 0.0318 for EC^ANGPT1^-SMC versus EC-SMC co-culture, *p = 0.0463 for EC^ANGPT1^-SMC^VEGF^ versus EC-SMC co-culture). **d** αSMA-wrapped vessels. Data are expressed as box-and-whisker plots, where the central lines denote medians, edges represent upper and lower quartiles and whiskers show minimum and maximum values. Data were analyzed by one-way ANOVA, followed by Holm–Sidak’s multiple comparison test, *n* = 5 (*p = 0.0444 for EC^ANGPT1^-SMC versus EC-SMC co-culture, **p = 0.0020 for EC^ANGPT1^-SMC^VEGF^ versus EC-SMC co-culture, ***p = 0.0003 for EC-SMC^VEGF^ versus EC-SMC co-culture, ***p = 0.0006 for EC-SMC^-^Myo versus EC-SMCco-culture, ****p < 0.0001 for EC^ANGPT1^-SMC^VEGF^-Myo versus EC-SMCco-culture)

Endothelial cell^ANGPT1^-bearing constructs fluorescently stained for α-smooth muscle actin (αSMA; Fig. 3a, b) demonstrated higher levels of total αSMA on day 4 post seeding compared to those bearing endothelial cell-containing cultures (Fig. 3c).

### ANGPT1 and VEGF promote mature vessel-like network formation

**Fig. 4:**
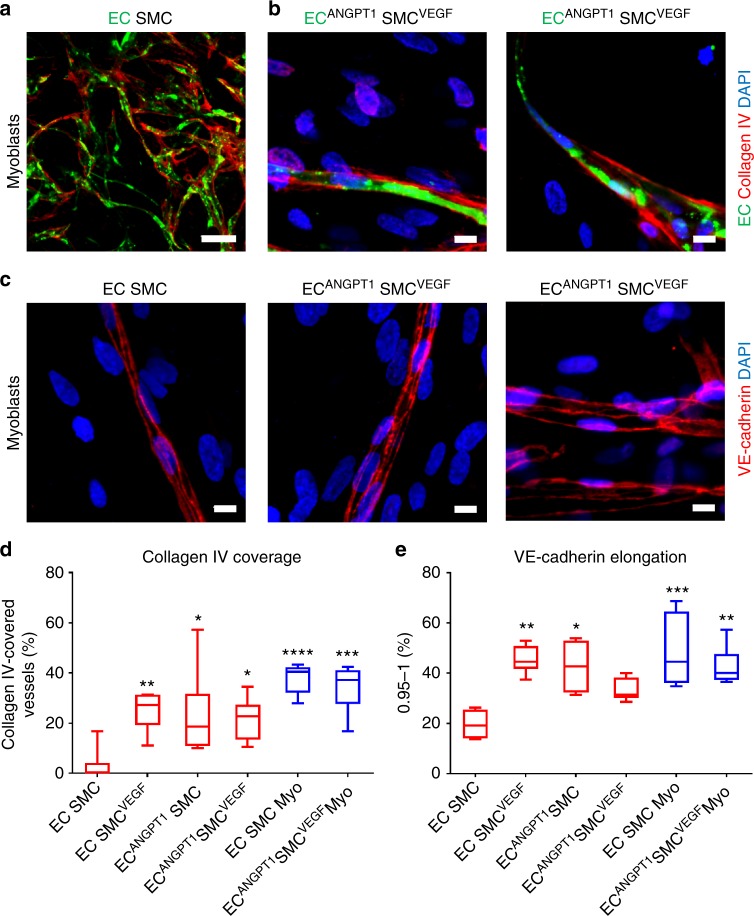
In vitro vessel-like network maturity. **a** Representative confocal images of whole-mount immunofluorescent scaffolds populated with tri-cultures. EC endothelial cells, SMC smooth muscle cells. Endothelial cell-ZsGreen are stained in green, collagen IV-positive cells are stained in red and nuclei are stained in blue; scale bar = 100 µm. **b** Representative confocal images of whole-mount immunofluorescent scaffold populated with endothelial cell^ANGPT1^-SMC^VEGF^-myoblast tri-culture. Endothelial cell^ANGPT1^-ZsGreen are stained in green, collagen IV-positive cells are stained in red and nuclei are stained in blue; scale bar = 10 µm. **c** Representative confocal images of whole-mount immunofluorescent scaffolds populated with co- or tri-cultures. Endothelial cell-ZsGreen are stained in green, VE-cadherin-positive cells are stained in red and nuclei are stained in blue; scale bar = 10 µm. **d** Collagen IV-wrapped vessels. Myo myoblasts. Data are expressed as box-and-whisker plots, where the central lines denote medians, edges represent upper and lower quartiles and whiskers show minimum and maximum values. Data were analyzed by one-way ANOVA, followed by Holm–Sidak’s multiple comparison test, *n* = 5 (*p = 0.0176 for EC^ANGPT1^-SMC versus EC-SMCs co-culture, *p = 0.0326 for EC^ANGPT1^-SMC^VEGF^ versus EC-SMC co-culture, **p = 0.0077 for EC-SMC^VEGF^ versus EC-SMC co-culture, ***p = 0.0001 for EC^ANGPT1^-SMC^VEGF^-Myo versus EC-SMC co-culture, ****p < 0.0001 for EC-SMC-Myo versus EC-SMC co-culture). **e** VE-cadherin-positive vessel elongation. Data are expressed as box-and-whisker plots, where the central lines denote medians, edges represent upper and lower quartiles and whiskers show minimum and maximum values. Data were analyzed by one-way ANOVA, followed by Holm–Sidak’s multiple comparison test, *n* = 5 (*p = 0.0119 for EC^ANGPT1^-SMC versus EC-SMC co-culture, **p = 0.0015 for EC-SMC^VEGF^ versus EC-SMC co-culture, **p = 0.0051 for EC^ANGPT1^-SMC^VEGF^-Myo versus EC-SMC co-culture, ***p = 0.0004 for EC-SMC^-^Myo versus EC-SMC co-culture)

The number of elongated endothelial cell vessels surrounded by αSMA-expressing mural cells (Fig. 3b and Supplementary Movie 1 and 2) was higher in cultures with either ANGPT1- or VEGF-expressing cells (Fig. 3d), suggesting the formation of stable vessels^23^. To further study the influence of ANGPT1 and VEGF overexpression on vessel maturation, the vascularized constructs were stained for collagen IV and vascular endothelial (VE)-cadherin (Fig. 4). Cultures containing either endothelial cell^ANGPT1^ or SMCs^VEGF^ demonstrated higher percentage of collagen IV-wrapped vessels (Fig. 4d and Supplementary Movie 3 and 4) and elongated VE-cadherin structures (Fig. 4e), as compared to naïve endothelial cell–SMC co-cultures.

### ANGPT1- and VEGF-expressing grafts promote neovascularization

**Fig. 5:**
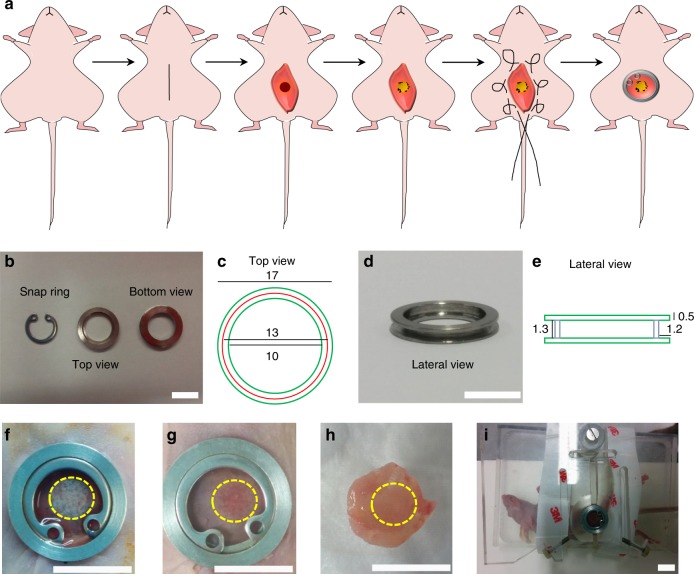
Images and schematic drawings of the AIW. **a** A schematic presentation of the surgical procedure. **b**, **c** Top and bottom view of the AIW. Scale bar = 10 mm. **d**, **e** Lateral view of the AIW. Scale bar = 10 mm. **f** The AIW immediately post implantation in the abdominal muscle. The area of the transplanted graft is indicated by a yellow dashed circle. Scale bar = 10 mm. **g** The AIW 14 days post implantation in the abdominal muscle. The area of the transplanted graft is indicated by a yellow dashed circle. Scale bar = 10 mm. **h** Graft integration 14 days post implantation in the abdominal muscle. The area of the transplanted graft is indicated by a yellow dashed circle. Scale bar = 10 mm. **i** Image of a mouse stabilized in a custom-made SID for intravital confocal imaging. Scale bar = 10 mm

**Fig. 6:**
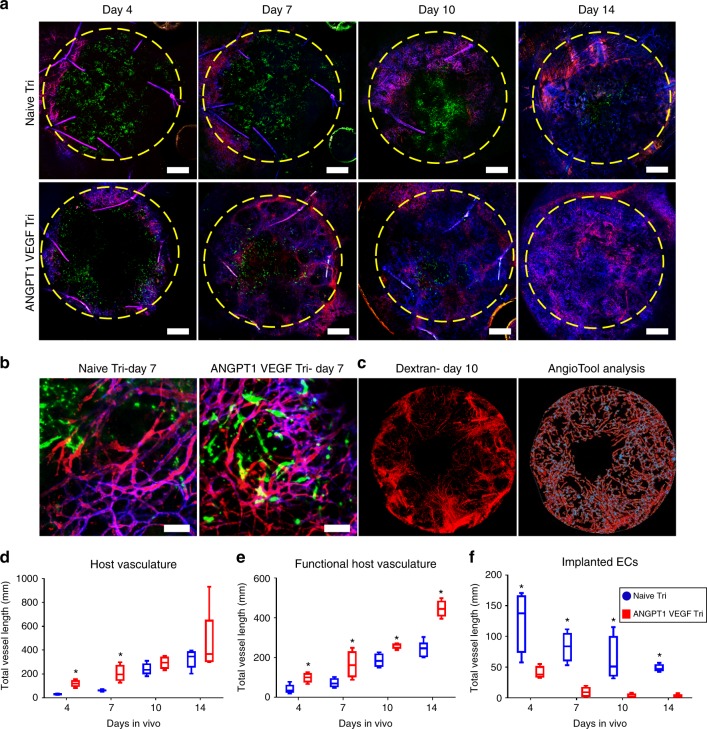
In vivo host neovascularization. **a** Representative intravital confocal images of the graft through the AIW. Naive tri-culture and ANGPT1- and VEGF-expressing tri-cultures were grown for 4 days prior to transplantation. Mice were injected with mCD31 to stain the host vessel network, and TRITC–dextran to observe perfusion. Green indicates human endothelial cells-ZsGreen, red indicates TRITC–dextran and blue indicates mouse CD31. The area of the transplanted graft is indicated by a yellow dashed circle. Scale bar = 100 µm. **b** Representative intravital confocal large-magnification images of grafts populated with naive or with ANGPT1- and VEGF-expressing tri-cultures 7 days post transplantation, viewed through the AIW. Green indicates human endothelial cell-ZGreen, red TRITC–dextran and blue mouse CD31. Scale bar = 100 µm. **c** Representative image of TRITC–dextran circulating in the transplanted scaffolds, and its respective AngioTool analysis. Vessel skeletons are seen in red, vessel borders in yellow and vessel junctions in blue. **d** AngioTool-quantitated total vessel length of both functional and nonfunctional mouse blood vessels. Data are expressed as box-and-whisker plots, where the central lines denote medians, edges represent upper and lower quartiles and whiskers show minimum and maximum values, *n* = 6. At each time point, unpaired two-tailed Student’s *t*-test analysis was performed between the tested groups (*p = 0.0012 and 0.0071 for ANGPT1 VEGF tri versus naive tri at days 4 and 7, respectively). **e** AngioTool-quantitated total vessel length of functional blood vessels. Data are expressed as box-and-whisker plots, where the central lines denote medians, edges represent upper and lower quartiles and whiskers show minimum and maximum values, *n* = 6. At each time point, unpaired two-tailed Student’s *t*-test analysis was performed between the tested groups (*p = 0.0089, 0.0426, 0.0088 and 0.00016 for ANGPT1 VEGF tri versus naive tri at days 4, 7, 10 and 14, respectively). **f** AngioTool-quantitated total vessel length of implanted endothelial cells (naive or ANGPT1-expressing). Data are expressed as box-and-whisker plots, where the central lines denote medians, edges represent upper and lower quartiles and whiskers show minimum and maximum values, *n* = 6. At each time point, unpaired two-tailed Student’s *t*-test analysis was performed (*p = 0.016, 0.0012, 0.019 and 0.0001 for ANGPT1 VEGF tri versus naive tri at days 4, 7, 10 and 14, respectively)

After establishing that ANGPT1-overexpressing endothelial cells increase both the length and the maturity of the vessel-like networks formed in vitro, we sought to examine their effect on host vascularization upon their transplantation into a rectus abdominis muscle defect of nude mice^12,15,21,24^ (Fig. 5a). Engineered muscles formed from a naive endothelial cell–SMC–myoblasts tri-culture, as a control, and from an endothelial cell^ANGPT1^, SMC^VEGF^ and myoblasts tri-culture were implanted 4 days post seeding. In both groups, highly effective host–graft integration was noticeable 14 days post transplantation (Fig. 5f, h). While host vessel invasion into both graft types was observable as early as 4 days post transplantation, its progression and coverage of the implanted grafts were faster in the ANGPT1- and VEGF-expressing grafts (Fig. 6). By 14 days post transplantation, ANGPT1- and VEGF-expressing grafts were more densely populated with host vasculature than the control tri-culture grafts, and the implanted vasculature was no longer visible. On days 4 and 7 post transplantation, total vessel length of the host vasculature was greater in ANGPT1- and VEGF-expressing grafts compared to control grafts (Fig. 6c, d). Concurrently, functional host vasculature was longer in ANGPT1- and VEGF-expressing grafts compared to control grafts at all tested time points (Fig. 6e). At each time point, implanted endothelial cells were noticeable mostly in the control grafts and were no longer visible at an earlier time point in the ANGPT1- and VEGF-expressing grafts compared to control (Fig. 6f). By day 14, host vessel density of both the control and the ANGPT1- and VEGF-expressing grafts was comparable with the physiological vessel density of the surrounding native muscle (Supplementary Fig. 2). Moreover, images of hematoxylin and eosin (H&E)-stained transplanted grafts clearly identified erythrocytes inside the invading host vessels (Supplementary Fig. 3), indicating that they were perfused and functional.

**Fig. 7:**
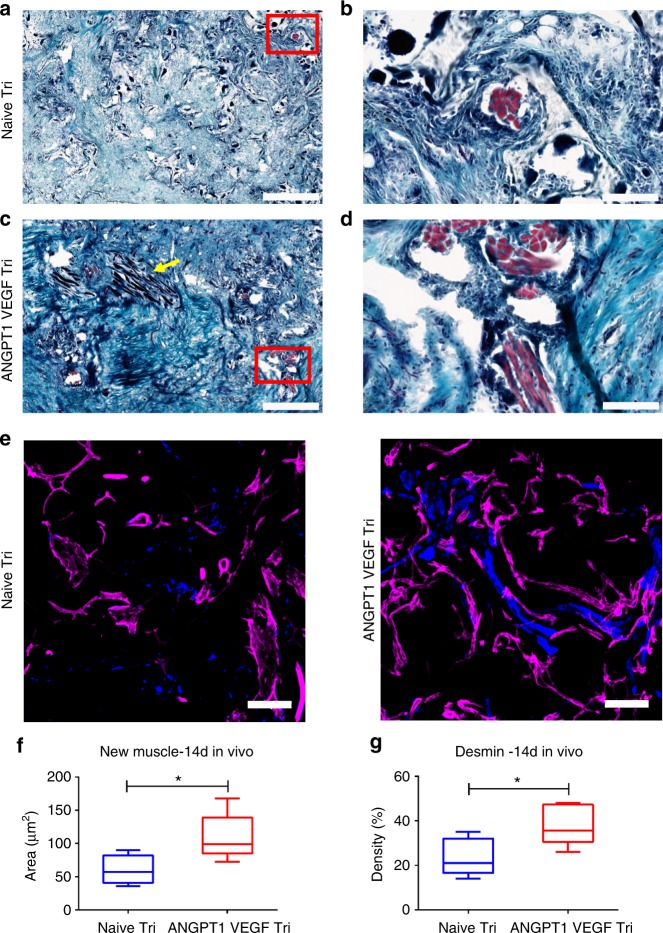
New muscle formation in vivo. **a** Representative images of Masson’s trichrome-stained naive tri-culture-embedded scaffolds 14 days post transplantation. Newly formed muscle bundles are seen in light purple. Scale bar = 500 µm. **b** Magnification of the red rectangle in (**a**). Scale bar = 100 µm. **c** Representative images of Masson’s trichrome-stained ANGPT1- and VEGF-secreting tri-culture-embedded scaffolds 14 days post transplantation. Newly formed muscle bundles are seen in light purple and a mature muscle in black is indicated by a yellow arrow. Scale bar = 500 µm. **d** Magnification of the red rectangle in (**c**). Scale bar = 100 µm. **e** Representative immunofluorescent signals of stained cryo-sections. Blue indicates desmin-positive staining, magenta mouse CD31-positive staining. Scale bar = 100 µm. **f** New myofiber area quantification. Data are expressed as box-and-whisker plots, where the central lines denote medians, edges represent upper and lower quartiles and whiskers show minimum and maximum values. Data analyzed by unpaired two-tailed Student’s *t*-test, *n* = 6 (*p = 0.0370). **g** Myofiber area quantification, as determined by desmin staining. Data are expressed as box-and-whisker plots, where the central lines denote medians, edges represent upper and lower quartiles and whiskers show minimum and maximum values. Data analyzed unpaired two-tailed Student’s *t*-test, *n* ≥ 4 (*p = 0.0268)

While young, desmin-positive, muscle fibers formed both around and inside the graft area within 14 days of transplantation of either graft type (Fig. 7), they covered a larger area in the ANGPT1- and VEGF-expressing grafts as compared to control grafts (Fig. 7f, g), indicating more effective myogenesis in the former.

## Discussion

Insufficient vascularization after implantation of engineered tissues can lead to transplantation failure with tissue necrosis. Therefore, prevascularization of thick implanted tissue is expected to enhance the chances of implanted tissue survival^9,11,12,15,17,24^. In this report, we combined gene therapy and tissue engineering techniques and showed that overexpression of ANGPT1 and VEGF in endothelial cells and SMCs co-cultured on 3D scaffolds enhanced maturity and length of the spontaneously forming vessel-like networks in vitro. This work also established the superior vasculogenic potential of the human endothelial cell^ANGPT1^, SMC^VEGF^ and myoblast tri-culture upon its transplantation into an abdominal wall defect in the nude mouse musculature, as compared to tri-cultures composed of naive cells only; scaffolds with genetically modified tri-cultures were fully vascularized by host vessels 14 days post transplantation. In addition, the overexpressing tri-culture scaffolds had more new muscle bundles compared to the naive tri-culture scaffolds, thus enhancing not only implantation but also regeneration. While this study focused on construction of a vascularized muscle tissue, the technique can be easily translated toward construction of other types of vascularized engineered tissues, by integrating a relevant tissue-specific cell type in the tri-culture.

The vasculogenic potential of cells isolated from limb veins of elderly patients undergoing bypass surgery has already been established^12^. As the present study aimed to optimize and accelerate the integration process, gene therapy techniques were employed in the construction of the vascularized engineered muscle. The choice of endothelial cells for expression of ANGPT1 and SMCs for expression of VEGF was set based on results obtained in collagen-embedded spheroid assay, demonstrating a twofold increase in dual endothelial cell and SMC sprouts compared to a combination of endothelial cells expressing VEGF and SMCs expressing ANGPT1^25^ (Supplementary Fig. 4). Intra-arterial co-injection of endothelial cell^ANGPT1^ and SMC^VEGF^ has been shown to promote arteriogenesis in animal models and was reported safe in phase 1 and 1b clinical studies treating patients with critical limb ischemia^19,20^. Here, we show that endothelial cell^ANGPT1^ and SMC^VEGF^ combination enhanced the vasculogenic potential of adult endothelial cells and SMCs in engineered tissue constructs as well, as compared to naive cells.

In our previous study monitoring implanted scaffolds embedded with a similar tri-cell combination^12^, graft integration was only monitored 9 days post transplantation into mice abdominal wall defect, limiting our understanding of integration dynamics. In the present study, the AIW enabled intravital imaging of the muscle tissue and host neovascularization throughout the 14-day post-implantation period, while significantly reducing the number of required animals. Most of the newly formed vessels within the transplant were epigastric vessels invading the transplant from the sides and hence appeared like an intact vascular network with a minimal granular pattern. However, some vessels extended perpendicular to the transplant and had a more scattered granular pattern. In most vessels, tetramethyl rhodamine isothiocyanate (TRITC)–dextran was located almost entirely inside the newly formed vessels, indicating minimal blood leakage. Nonetheless, some degree of vascular leakage was detected, as expected of a normal wound healing process^26^. In light of earlier work demonstrating that overexpression of ANGPT1 and VEGF in mice resulted in leakage-resistant vessels^27^, it is likely that the blood vessels formed within the ANGPT1- and VEGF-secreting muscle are less leaky. The analysis revealed longer host vessels in the genetically modified tri-culture versus control naive tri-culture scaffolds on days 4 and 7 post transplantation (Fig. 6). In parallel, the number of TRITC–dextran-perfused functional host vessels penetrating the transplanted graft was higher at all tested time points in the modified tri-culture group compared to the control group (Fig. 6). We noted rapid replacement of the engineered blood vessels by the host vessels, indicative of fast vascular remodeling of the transplant. The newly formed vessels were perfused and functional, as indicated by TRITC–dextran perfusion and erythrocyte presence inside the invading host vessels (Fig. 6 and Supplementary Fig. 3). Replacement of the implanted endothelial cell^ANGPT1^ versus endothelial cells with host vessels was faster (Fig. 6). Koffler et al.^24^ also demonstrated that more advanced endothelial cell organization in vitro resulted in faster anastomosis and replacement by the host blood vessels. They suggest that rapid replacement is advantageous, providing an immediate platform for remodeling of the blood vessel network to host needs. The present set-up is favorable in its exploitation of cells readily isolatable from the patient’s short vein (endothelial cells and SMC) or from a small muscle biopsy (myoblasts). Furthermore, endothelial cells and SMCs of such sources have been fully characterized^19,20,25,28^ and their isolation, ANGPT1 and VEGF transduction and use in intra-arterial injection into critical limb ischemia patients is seemingly safe^19,20^.

The presented findings demonstrate the clear benefit of using engineered muscle that secretes both ANGPT1 and VEGF, as compared to naive constructs. Elongated vessel-like structures were spontaneously formed in vitro in co- and tri-cultures containing endothelial cell^ANGPT1^ (Fig. 2). SMCs^VEGF^ cultured with naive endothelial cells failed to induce a significant effect on elongation of the vessel-like networks in vitro (Fig. 2). However, overexpression of either VEGF by SMCs or ANGPT1 by endothelial cells resulted in an increase in vessel stability and maturation, as determined by SMC-, collagen IV- and VE-cadherin-vessel coverage (Figs. 3d and 4 and Supplementary Movie 1−4). Although VE-cadherin junctional labeling appeared elongated in all co- and tri-culture constructs overexpressing ANGPT1 and/or VEGF, the junctional patterning in the co-cultures appeared weaker and serrated, indicative of angiogenic vessels. With the addition of myoblasts, the straight, parallel VE-cadherin junctional line patterning was indicative of mature vessels (Fig. 4c)^29,30^. The expression of collagen IV was also enhanced in the tri-cultures. Collagen IV expression is localized to mature vessels and is not found around immature vascular sprouts^31,32^. VEGF is crucial for endothelial cell sprouting, angiogenesis and maintenance of the quiescent phalanx resolution^33–35^. ANGPT1 promotes pericyte recruitment and maturation, as well as maintenance of the quiescent phalanx resolution^33,34^. Gluzman et al.^25^ showed that co-culture of adult autologous endothelial cell^ANGPT1^ with adult autologous SMCs^VEGF^ resulted in coordinated vascular sprouting using collagen-embedded spheroids assay in vitro and increased arteriogenesis in vivo upon intra-arterial injection. Similarly, we noted promotion of host neovascularization (Fig. 6) and enhanced myogenesis (Fig. 7) in the presence of this cell combination within engineered muscle tissue, as compared to the control. The myogenesis identified in the transduced tri-culture grafts (Fig. 6) was ascribed to enhanced VEGF secretion from the graft, as previous works have shown that VEGF administration induces muscle regeneration^36–40^. We have previously demonstrated that even minimal VEGF secretion from myoblasts upon implantation of constructs containing endothelial cells, myoblasts and supporting cells, resulted in substantial muscle regeneration involving satellite cell activation, proliferation, differentiation, fusion to myofibers, and maturation^21,24^. Hence, enhanced muscle regeneration is expected with increased VEGF secretion from the transplanted grafts. Muscle functionality will likely be enhanced in the transduced tri-culture grafts, as we have previously established that improved vascularization leads to improved muscle functionality^24^. Host vessel density within both the control and the ANGPT1- and VEGF-expressing grafts was comparable with the physiological vessel density of the surrounding native muscle (Supplementary Fig. 2), suggesting that the vascular density of the transplant is sufficient to support normal levels of muscle activity.

Here, we constructed, for the first time to our knowledge, a genetically engineered vascularized muscle composed entirely from human cells over-secreting ANGPT1 and VEGF. The described vascular cells can be easily isolated from elderly patients and used to pre-vascularize various tissue types, ultimately constructing autologous vascularized tissues that promote neovascularization and integration within the host. Although the described primary cells are isolated from patients with advanced arterial disease, they retained their capacity to proliferate under the tested conditions^25^. The use of vascular cells derived from the elderly, is of great importance, since they can be applied for the construction of autologous tissues for adult patients, without the issue of rejection. By employing gene therapy in the construction of the vascularized engineered muscle, we successfully optimized and accelerated the integration process. Since the utilized vascular cells are fully differentiated with no telomerase activity, they are not likely to undergo oncogenic transformations after gene transfer^19^. However, the feasibility of our proposed autologous therapy is not without disadvantages. First, there are many regulatory challenges that present along the bench to bedside route of autologous engineered tissue products^41^ and secondly, the culturing time of the harvested cells is relatively long and entails many expenses. In summary, the present study demonstrated successful reconstruction of an abdominal wall defect in nude mice, by muscle grafts genetically modified to secrete both VEGF and ANGPT1. The described engineered construct has the potential to be fully translated to the clinic setting to overcome autologous flap shortage and to accelerate host neovascularization and integration of engineered grafts following transplantation. However, further efforts are still required to construct larger tissues that can be utilized in the clinic. Rosenfeld et al.^42^ have shown that aligned and oriented vascular networks implanted in the direction of muscle fibers in the mouse abdominal muscle, integrated more effectively than nonaligned control samples, demonstrating the need to match the implanted engineered tissue structure with the host tissue structure. Thus, the structure of the engineered muscle will have to be optimized to better mimic that of native muscle tissue. Tri-cultures with myoblasts were used as a proof of concept to demonstrate that vessel networks can also form within skeletal muscle. Nevertheless, other types of tissue-specific cells should be tested to show the broad applicability of this model. Lastly, before translation of these vascularized engineered grafts into clinical use, comparison of endothelial cells and SMCs from numerous donors and transplantation into larger animal models will still be necessary.

## Methods

### Cell culture

Adult primary venous endothelial cells and adult primary venous smooth muscle cells (adult SMCs) were isolated from a lower-extremity vein segment of elderly patients undergoing bypass surgeries, as previously described^19,25,28^. ANGPT1- and ZsGreen fluorescent protein-expressing adult venous endothelial cells (endothelial cell^ANGPT1^) were cultured in endothelial basal growth medium-2 supplemented with fibroblast growth factor-2, insulin-like growth factor-1 and epidermal growth factor from the EGM-2 BulletKit (Lonza, USA), and 3% fetal bovine serum (FBS; HyClone, Thermo Fisher Scientific, USA). Both VEGF-expressing (SMC^VEGF^) and naive adult SMCs were cultured in Dulbeccos modified Eagles medium, supplemented with 20% FBS and penicillin/streptomycin solution. The use of vein segments not utilized in surgery was approved by the Lady Davis Carmel Medical Center Review Board. Informed consent was obtained from the patients scheduled to undergo bypass surgery. Primary human skeletal muscle cells (hMyo) were purchased from PromoCell and cultured in the recommended PromoCell skeletal muscle cell growth medium. Cells were incubated in a 5% CO_2_ humidified atmosphere at 37 °C. Cells were split (1:3) for sub-culturing and used at passage 8 or 9 for all experiments.

### Lentivirus and retroviral transduction

All endothelial cells utilized in this study were pre-labeled with untargeted fluorescent protein by ZsGreen lentivirus particles, as previously described^12^. For endothelial cell^ANGPT1^, adult endothelial cells were first transduced with a retroviral vector to express ANGPT1 and then transduced with ZsGreen lentivirus particles. Adult SMCs were transduced with a retroviral vector to express VEGF_165_ (SMC^VEGF^), as previously described^19,43^. Transgene expression was determined by immunohistochemistry (IHC) and ELISA. Minimal IHC expression levels were set to 55% for endothelial cells expressing ANGPT1 and 60% for SMCs expressing VEGF_165._ The minimal transgene expression level by ELISA was 0.05 pg for ANGPT1 and VEGF_165_ protein/single cell/24 h^20^. These minimal levels were set based on results obtained in efficacy studies in animals and on in vitro sprouting studies^25^. Endothelial cell^ANGPT1^–SMC^VEGF^ combination was set based on results obtained in collagen-embedded spheroid assay (Supplementary Fig. 4).

### PLLA/PLGA scaffold fabrication

The salt-leaching technique was utilized to fabricate 3D porous poly-l-lactic acid (PLLA) (Polysciences, Warrington) and polylactic glycolic acid (PLGA; Boehringer Ingelheim) (1:1) scaffolds, with pore sizes of 212−600 µm and 93% porosity, as previously described^12,15,21,44^. Briefly, a 1:1 polymer solution of PLLA and PLGA dissolved in chloroform was prepared. Next, 0.24 ml of this solution was added to 0.4 g sodium chloride particles in 18 mm-diameter Teflon molds. The chloroform was allowed to evaporate overnight and the scaffolds were washed in distilled water for 8 h. Before each experiment, the 1 mm-thick scaffolds were cut into 6 mm-diameter circles, soaked in 70% (v/v) ethyl alcohol for 2 h and then washed 3 times with phosphate-buffered saline (PBS).

### Engineering vascularized 3D constructs

Six multicellular cultures were examined in this study: (i) endothelial cells (0.5 × 10^6^ cells) and SMCs (0.1 × 10^6^ cells) were cultured to generate 3D vascular networks; (ii) endothelial cells (0.5 × 10^6^ cells) and SMCs^VEGF^ (0.1 × 10^6^ cells) were cultured to generate 3D vascular networks; (iii) endothelial cell^ANGPT1^ (0.5 × 10^6^ cells) and SMCs (0.1 × 10^6^ cells) were cultured to generate 3D vascular networks; (iv) endothelial cell^ANGPT1^ (0.5 × 10^6^ cells) and SMCs^VEGF^ (0.1 × 10^6^ cells) were cultured to generate 3D vascular networks; (v) endothelial cells (0.5 × 10^6^ cells), SMCs (0.1 × 10^6^ cells) and hMyo (0.2 × 10^6^ cells) were cultured to generate 3D vascular networks within skeletal muscle constructs; and (vi) endothelial cell^ANGPT1^ (0.5 × 10^6^ cells), SMCs^VEGF^ (0.1 × 10^6^ cells) and hMyo (0.2 × 10^6^ cells) were cultured to generate 3D vascular networks within skeletal muscle constructs.

Co- and tri-culture cell ratios and concentrations were determined based on previous studies^15,21,24^. Cells were trypsinized at passage 8 or 9 and re-suspended in 4 µl of a 5 U/ml thrombin solution (Johnson & Johnson Medical, Israel). Then, 4 µl of 15 mg/ml fibrinogen (Johnson & Johnson Medical, Israel) was added to the cell–thrombin mixture and each cell suspension was seeded onto the PLLA/PLGA scaffolds and then allowed to solidify for 30 min (37 °C, 5% CO_2_) inside a 6-well non-tissue culture plate. After solidification, 4 ml culture medium was added to each well. Medium was replaced every other day.

### Vessel network elongation assessment

To evaluate the elongation of the vessel-like structures formed by various cell combinations in vitro, scaffolds were imaged, with a confocal microscope, 4 and 7 days post seeding and eccentricity of ZsGreen-positive vessel-like structures was calculated using a self-written MATLAB (MATLAB^©^, MathWorks) algorithm. Briefly, each *Z*-stack image was transformed into a binary image, and then separated into distinct elements before eccentricity was determined using the “regionprops” function. A circle-shaped element received a value of zero and more elongated elements received an eccentricity value closer to one (Supplementary Fig. 1 and Supplementary Software 1 to 4).

### Whole-mount immunofluorescence staining

Whole, cell-embedded scaffolds were stained as previously described^12^. First, scaffolds were fixated in 4% paraformaldehyde (Electron Microscopy Sciences, USA) for 10 min and subsequently washed with PBS. Next, the cells were permeabilized by adding 0.3% Triton X-100 (Bio Lab Ltd.) for 10 min at room temperature (RT). The scaffolds were then washed with PBS and soaked for 1 h in blocking serum (10% FBS, 0.1% Triton X-100, 1% glycine) at RT. Subsequently, scaffolds were incubated overnight at 4 °C with the following primary antibodies (diluted in blocking solution): mouse anti-human αSMA (1:50, Dako), mouse anti-human collagen IV (1:500, Sigma-Aldrich) and mouse anti-human VE-cadherin (1:150, Santa Cruz). Following four washes of 10 min each in PBS, Cy3-conjugated donkey anti-mouse IgG (1:100, Jackson Immuno-research laboratory, PA) was applied for 3 h. Nuclei were counterstained with 4’,6-diamidino-2-phenylindole (DAPI) (1:1000, Sigma-Aldrich). Scaffolds were then washed in PBS and stored in 24-well plates in PBS at 4 °C until observation under a Zeiss LSM700 inverted confocal microscope (Carl Zeiss), with 5× and 10× objective lenses, using ZEN software (Carl Zeiss). Further image analysis was conducted using FIJI (Fiji Is Just ImageJ) software.

### Vessel stability and maturity determination

To evaluate vessel stability and maturity, scaffolds were stained for αSMA, collagen IV and VE-cadherin. Scaffolds were imaged with a confocal microscope 4 and 7 days post seeding and *z*-stack confocal projection images of the scaffolds were analyzed.

Total area of αSMA expression was quantified using a self-written MATLAB algorithm.

Elongation of VE-cadherin-positive vessels was quantified using a self-written MATLAB algorithm for eccentricity determination (Supplementary Fig. 1 and Supplementary Software 1 to 4). The percentage of collagen IV- and αSMA-positive vessels was determined by an independent expert blinded to the culture components.

### ANGPT1, ANGPT2 and VEGF secretion determination

Supernatants from all scaffold groups were collected 4 days post seeding, and ANGPT1, ANGPT2 and VEGF concentrations were determined using human ANGPT1, ANGPT2 and VEGF ELISA detection kits (R&D SYSTEMS), respectively. The optical density (OD) of growth medium without cultured cells served as a control and was subtracted from the OD values of the tested groups.

### Abdominal imaging window

An AIW was developed by us by modifying the protocol described by Ritsma et al.^45,46^ and manufactured to suit live muscle imaging. The AIW consisted of a reusable stainless steel ring (17 mm outer diameter, 13 mm inner diameter, and 2.3 mm thick), with a 1.3 mm groove on the side (Fig. 6). After implantation, the AIW was sealed with a 13 mm circular glass coverslip coated with poly-L-lysine-g-poly (ethylene glycol). The coverslip was fixed in place with a snap ring, for subsequent intravital microscopy (Fig. 5).

### Stabilizing imaging device

A polymethyl methacrylate stabilizing imaging device (SID) was developed by us (Fig. 5i) to stabilize the mouse during intravital imaging and to minimize breathing movements, providing for focused images.

### Transplantation of the engineered tissue

All surgical procedures were conducted according to protocols approved by the Institutional Animal Care and Use Committee of the Technion IIT. Sample size was determined using power analysis (β = 0.1 and α = 0.05). Standardized effect size was calculated based on previous studies and a pilot experiment; the resulting sample size was 6. Four animals were excluded from the study since their AIW fell off prior to the end point. Two independent experiments were conducted and a minimum of three animals per treatment group were analyzed in each experiment. Selection of animals for the allocation to experimental and the control groups was randomized. Tri-cultures of adult endothelial cells, SMC and hMyo (*n* = 6) and of adult endothelial cell ^ANGPT1^, SMC ^VEGF^ and hMyo (*n* = 6) were cultured on PLLA/PLGA scaffolds for 4 days before implantation. Female, nude, 9-week-old mice (Harlan Laboratories) were anesthetized via intra-peritoneal injection of a ketamine–-xylazine mixture (100 mg/kg and 10 mg/kg, respectively), using a 30-gauge needle. Buprenorphine (0.05 mg/kg) was subcutaneously injected 20 min before the procedure and every 12 h thereafter for 3 days. The planned incision site was cleaned with alcohol and iodine to establish an aseptic working field. Then, the abdominal wall was exposed by a ventral skin incision, and a 6 mm-diameter full-thickness segment of the rectus abdominis muscle was removed. The engineered skeletal muscle graft was sutured in place using 8-0 polypropylene sutures.

### Abdominal imaging window implantation

Before use, the AIW was soaked overnight in 70% (v/v) ethyl alcohol and washed three times with PBS. AIW implantation was performed based on the protocol presented by Ritsma et al.^45,46^ with some modifications. Briefly, after suturing the engineered tissue in the abdominal wall, a purse-string suture was made around the incision through the skin, using 4-0 silk sutures. Next, cyanoacrylate glue was placed on the interior ring surface of the AIW and the AIW was glued around the transplanted tissue by applying gentle pressure. The skin was carefully placed in the groove of the AIW. Then, the loops of the purse-string suture were pulled, one by one, tightening the suture in the groove of the AIW. Then, a circular glass coverslip was placed on top and fixed by a snap ring. All mice were monitored closely for one to 2 h to ensure full recovery from the anesthesia. The mice were monitored daily to assess general health and were subsequently included in intravital microscopy studies.

### Intravital imaging

Intravital microscopy was performed 4, 7, 10 and 14 days post transplantation using an LSM700 confocal microscope. To visualize the host vasculature in mice, Alexa Fluor® 647-conjugated anti-mouse CD31 antibody (mCD31-X647; Biolegend) (0.5 mg/ml) was intravenously injected into the tail vein and allowed to circulate for ~15 min. Then, the mice were anesthetized (using ketamine–xylazine, as described above) and TRITC–dextran (10 mg/ml) (average molecular weight 155,000, Sigma-Aldrich) was intravenously injected into the tail vein to visualize functional vessels. Mice were placed in the SID and intravital microscopy was performed. The temperature was maintained at 28 °C, with a heating chamber, throughout the imaging session. At the end point, mice were killed and the grafts were retrieved and fixed in 10% formalin (Sigma-Aldrich).

### Vessel length quantification

Total length (mm) of vessels within 14-day post-transplantation constructs was calculated by analyzing *z*-stack confocal projection images using the AngioTool software (AngioTool^®^)^47^. Two independent experiments were conducted and a minimum of three animals per treatment group were analyzed in each experiment.

### Immunohistochemical and immunofluorescence staining

Grafts retrieved 14 days post transplantation were incubated overnight in a 30% (w/v) sucrose solution, embedded in optimal cutting temperature (OCT) compound (Tissue-Tec, USA), and frozen for subsequent cryosectioning to 5 and 10 μm-thick sections, as previously described^12^. Briefly, for immunofluorescence staining, the sections were incubated in 0.5% Tween solution for 20 min, rinsed with PBS and then blocked with a 5% (w/v) bovine serum albumin solution (Sigma-Aldrich) for an additional 30 min. Subsequently, sections were incubated overnight at 4 °C with goat anti-desmin (1:50, Santa Cruz) diluted in blocking solution. Next, sections were washed with PBS and labeled with Alexa 405-conjugated IgG (1:400; Molecular Probes) before being mounted in Fluromount-G (Southern Biotechnology) and examined under a confocal microscope. For H&E staining, slides were air-dried for several minutes, and stained with filtered 0.1% Mayers Hematoxylin (Sigma-Aldrich), for 10 min. Then, the slides were rinsed with tap water and stained with 0.5% Eosin (Sigma-Aldrich) for 1 min. Next, the slides were dipped in double distilled water (DDW) and dehydrated by serial immersions in increasing concentrations of ethanol. Lastly, slides were dipped in xylene and covered with Vectamount (Vector Labs). For Masson trichrome staining, the slides were air-dried and then stained with filtered 0.1% Mayers Hematoxylin for 5 min. Next, the slides were rinsed with distilled water and stained with trichrome stain (Sigma-Aldrich) for 2 min. Then, the slides were washed twice in 0.2% glacial acetic acid and then in DDW. Afterwards, the slides were dehydrated by serial immersions in increasing concentrations of ethanol, and finally dipped in xylene and covered with Vectamount. Slides were imaged with a Pannoramic MIDI automatic digital slide scanner (3DHISTECH, Hungary). Images were analyzed using the Pannoramic Viewer software (3DHISTECH, Hungary).

### Ex vivo vessel density quantification

Host vessel densities within grafts, retrieved 14 days post transplantation, and within the surrounding native muscle, were calculated by analyzing *z*-stack confocal projection images, using the FIJI software. Two independent experiments were conducted and a minimum of three animals per treatment group were analyzed in each experiment.

### Statistical analysis

Statistical analyses were performed using a computerized statistical program (GraphPad Software, Inc.). Data are presented in box-and-whisker plots, where the central lines denote medians, edges represent upper and lower quartiles and whiskers show minimum and maximum values. Group differences were determined by unpaired two-tailed Student’s *t*-test. Where appropriate, data were analyzed by one-way analysis of variance (ANOVA), followed by Holm–Sidak’s multiple comparison test. The *p* values below 0.05 were taken to indicate a statistically significant difference between groups.

### Code availability

MATLAB source code for vessel network elongation assessment is provided as Supplementary Software 3.

## Electronic supplementary material

Supplementary Information

Description of Additional Supplementary Files

Supplementary Movie 1

Supplementary Movie 2

Supplementary Movie 3

Supplementary Movie 4

Supplementary Software 1

Supplementary Software 2

Supplementary Software 3

Supplementary Software 4

## Data Availability

All the relevant data that are not in the article or in the Supplementary material are available from the corresponding author on request.
