# Supplementary material for: Genetically engineered human muscle transplant enhances murine host neovascularization and myogenesis
Source: Commun Biol. 2018 Oct 4;1:161. doi: 10.1038/s42003-018-0161-0 (PMC6172230; doi:10.1038/s42003-018-0161-0)
Supplement: Supplementary file 1 — Supplementary Information [file 42003_2018_161_MOESM1_ESM.pdf]

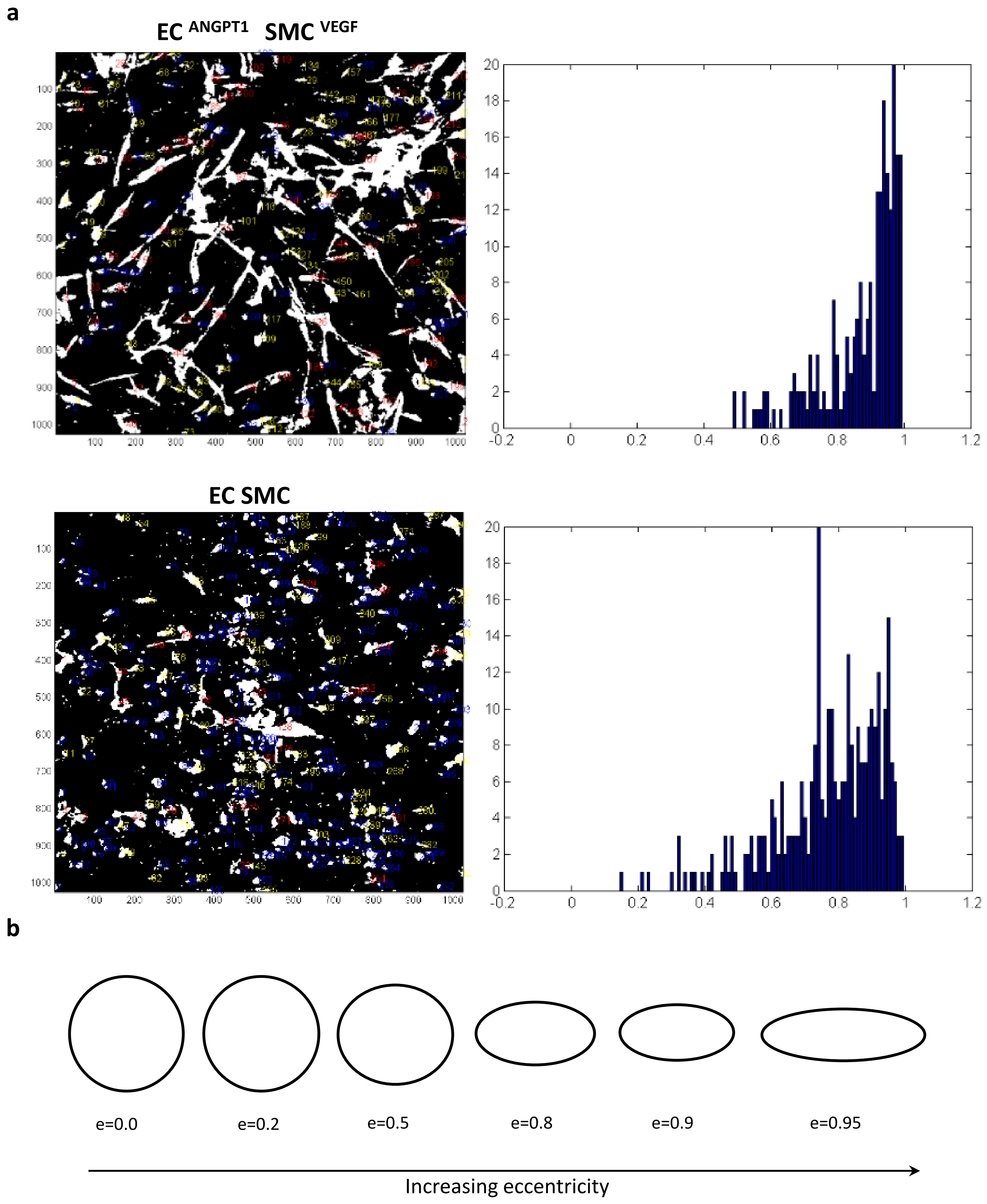

Supplementary Figure 1. In vitro vessel-like network eccentricity analysis. (**a**) Representative binary images of vessel-like networks of different eccentricity score profiles. Images were divided into elements and each element was scored according to its eccentricity. Data for each image are presented in the histograms. (**b**) Eccentricity scale.

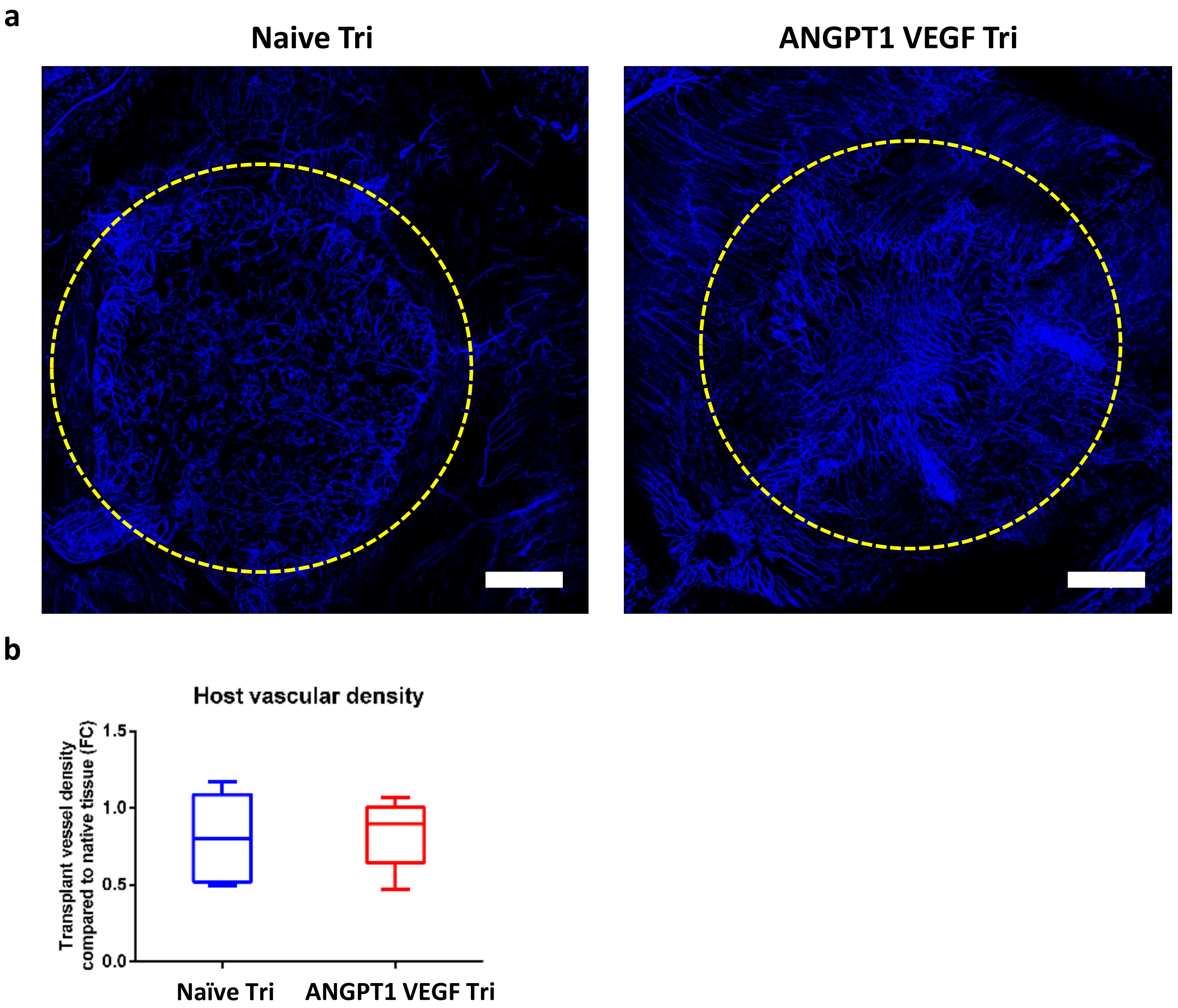

Supplementary Figure 2. Vascular density in graft vs. native muscle. (**a**) Representative confocal images of host vessels within the inner side of the grafts, 14 days post-transplantation. Naïve tri-culture and ANGPT1- and VEGF-expressing tri-cultures were grown for 4 days prior to transplantation. Blue - mouse CD31. The area of the transplanted graft is indicated by a yellow dashed circle. Scale bar = 1000 µm. (**b**) FIJI–quantitated fold change (FC) of host vessel density within the transplant (inside the yellow dashed circle) compared to the surrounding native muscle. Data are expressed as box-and-whisker plots, where the central lines denote medians, edges represent upper and lower quartiles and whiskers show minimum and maximum values, n=6.

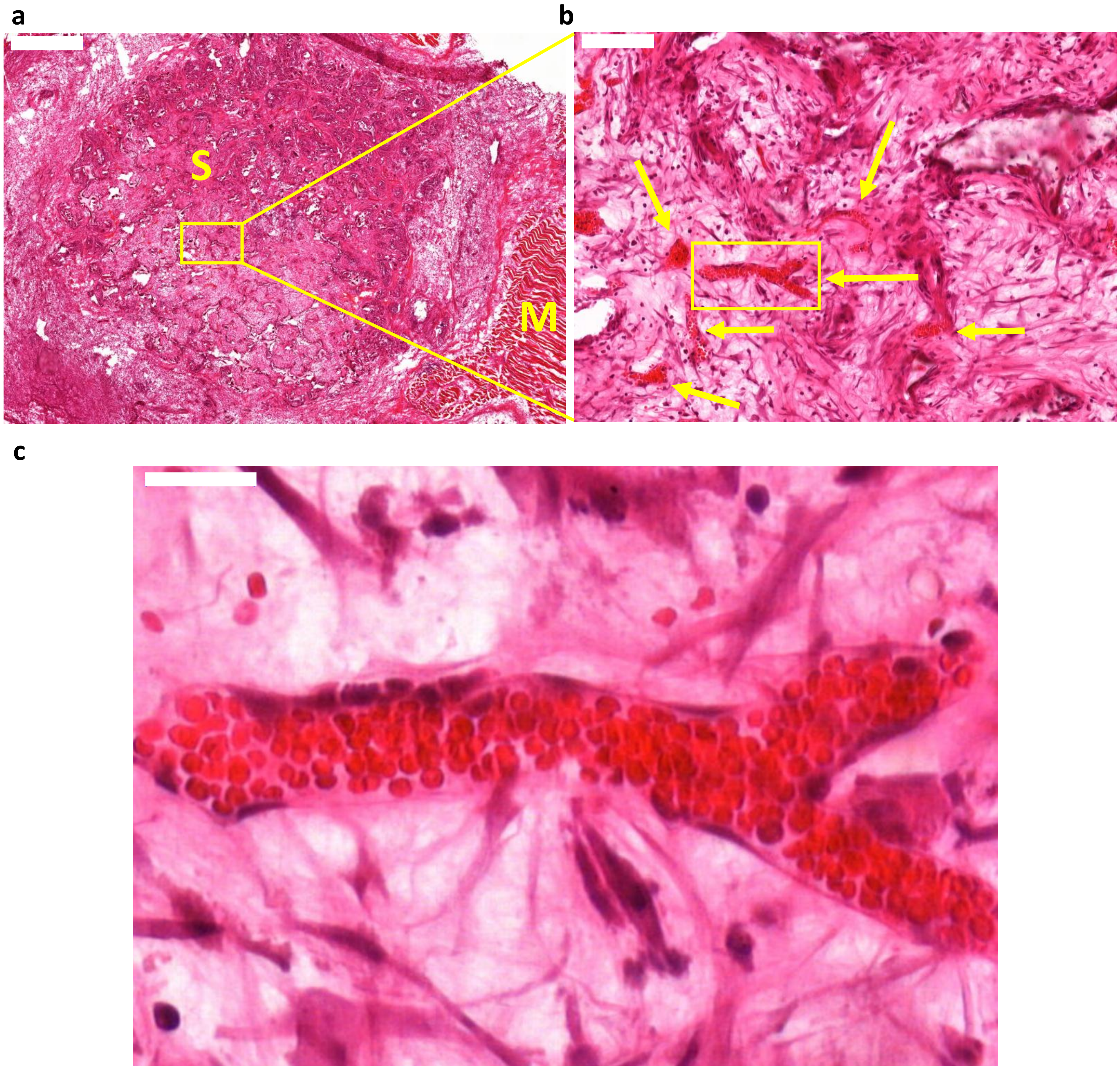

Supplementary Figure 3. Representative ex vivo graft images. (a) Representative image of a H&E-stained ANGPT1- and VEGF-expressing graft retrieved 14 days post-implantation. S: scaffold; M: native mouse muscle tissue. Scale bar = 1000 µm. **(b)** Large magnification of H&E-stained functional blood vessels containing erythrocytes in the center of the implanted scaffold. Erythrocyte-containing blood vessel is indicated by an arrow. Scale bar =100 µm. **(c)** Large magnification of H&E-stained functional blood vessels containing erythrocytes in the center of the implanted scaffold. Scale bar =20 µm.

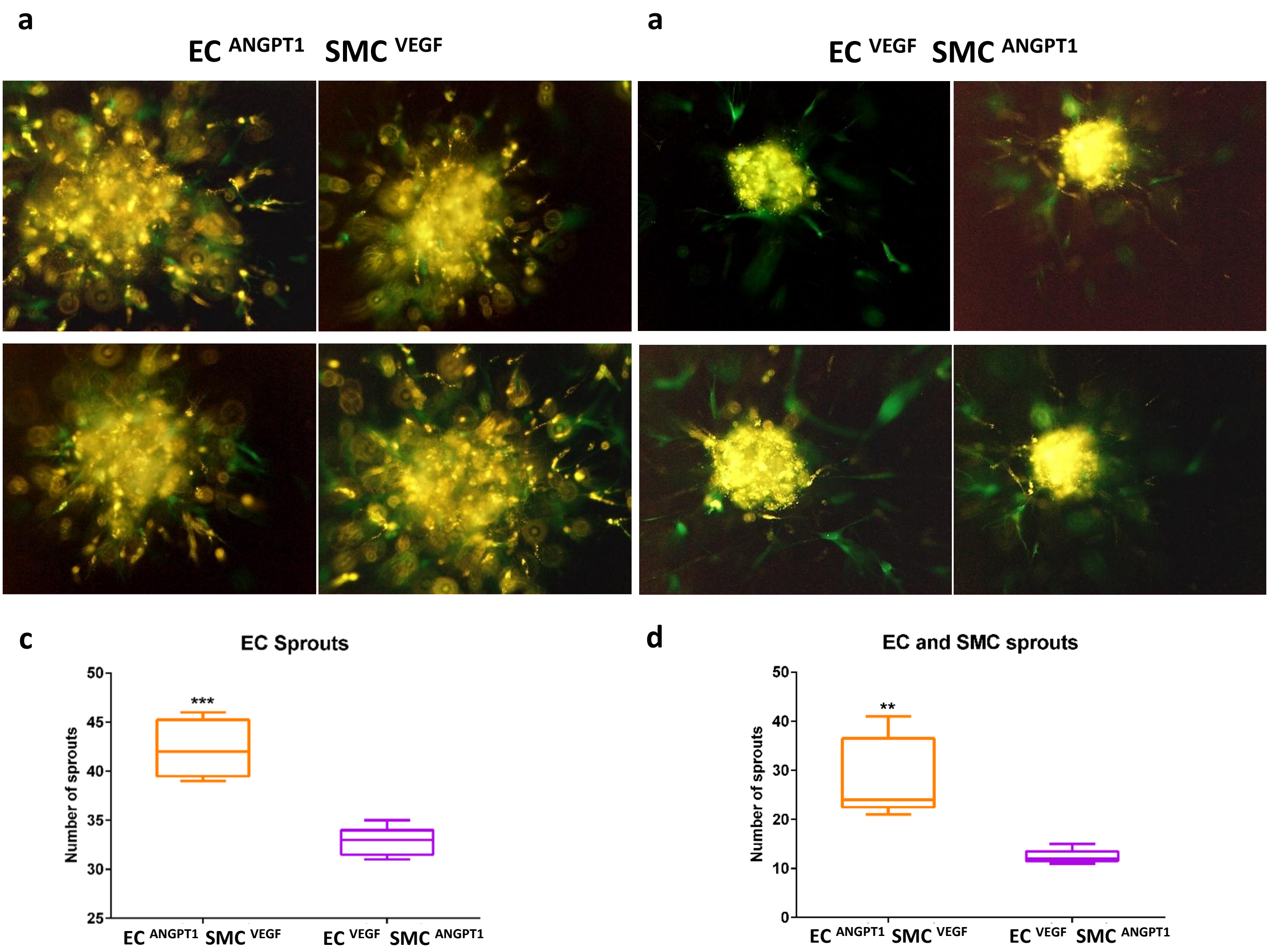

Supplementary Figure 4. Three-dimensional in-vitro angiogenesis spheroids assay. A mixture of 375 ECs and 375 SMCs were used to generate spheroids that were rapidly transferred into 24-well plates containing collagen and allowed to polymerize. The gels were incubated at 37°C and imaged 72 h post-seeding, using a digital camera (DXM1200 Nikon, Japan). ECs are presented in yellow and SMCs in green. **(a)** Representative image of spheroids comprised of EC over-expressing ANGPT1 and SMC over-expressing VEGF implanted in a three-dimensional collagen matrix. **(b)** Representative image of spheroids comprised of EC over-expressing VEGF and SMC over-expressing ANGPT1 implanted in a three-dimensional collagen matrix. **(c)** Quantification of number of EC sprouts. Data are expressed as box-and-whisker plots, where the central lines denote medians, edges represent upper and lower quartiles and whiskers show minimum and maximum values, n=5 (***p=0.0004). **(d)** Quantification of number of dual EC and SMC sprouts. Data are expressed as box-and-whisker plots, where the central lines denote medians, edges represent upper and lower quartiles and whiskers show minimum and maximum values, n=5 (**p=0.0025).
