# Supplementary material for: Genetically engineered human muscle transplant enhances murine host neovascularization and myogenesis
Source: Commun Biol. 2018 Oct 4;1:161. doi: 10.1038/s42003-018-0161-0 (PMC6172230; doi:10.1038/s42003-018-0161-0)
Supplement: Supplementary file 2 — Description of Additional Supplementary Files [file 42003_2018_161_MOESM2_ESM.docx]

**Description of Additional Supplementary Files**

File Name: Supplementary Movie 1

**Description: Three-dimensional reconstruction of αSMA-wrapped vessels shown in Figure 3B.** EC-ZsGreen are labeled in green, αSMA-positive cells are stained in red and nuclei are stained in blue. Volume rendering reconstruction and animation were performed on the z-series shown in Figure 3B using the ZEN software (Carl Zeiss). Experimental conditions were as described in the Figure 3B legend and Materials and Methods.

File Name: Supplementary Movie 2

**Description: Confocal z-slices movie of αSMA-wrapped vessels shown in Figure 3B.** EC-ZsGreen are labeled in green, αSMA-positive cells are stained in red and nuclei are stained in blue. Experimental conditions were as described in the Figure 3B legend and Materials and Methods.

File Name: Supplementary Movie 3

**Description: Three-dimensional reconstruction of collagen IV-wrapped vessels shown in Figure 4A.** EC-ZsGreen are labeled in green, collagen IV -positive cells are stained in red and nuclei are stained in blue. Volume rendering reconstruction and animation were performed on the z-series shown in Figure 4A using the ZEN software (Carl Zeiss). Experimental conditions were as described in the Figure 4A legend and Materials and Methods.

File Name: Supplementary Movie 4

**Description: Confocal z-slices movie of collagen IV-wrapped vessels shown in Figure 4A.** EC-ZsGreen are labeled in green, collagen IV-positive cells are stained in red and nuclei are stained in blue. Experimental conditions were as described in the Figure 4A legend and Materials and Methods.

File Name: Supplementary Software 1

**Description: A README file for vessel network elongation assessment.**

File Name: Supplementary Software 2

**Description: An image to demo the eccentricity MATLAB algorithm for vessel network elongation assessment.**

File Name: Supplementary Software 3

**Description: Eccentricity MATLAB algorithm for vessel network elongation assessment.**

File Name: Supplementary Software 4

**Description: Expected output for eccentricity MATLAB algorithm for vessel network elongation assessment.**
