# Supplementary material for: Genetically engineered human muscle transplant enhances murine host neovascularization and myogenesis
Source: Commun Biol. 2018 Oct 4;1:161. doi: 10.1038/s42003-018-0161-0 (PMC6172230; doi:10.1038/s42003-018-0161-0)
Supplement: Supplementary file 7 — Supplementary Software 1 [file 42003_2018_161_MOESM7_ESM.docx]

1. System requirements

**All software dependencies and operating systems (including version numbers):**

Matlab R2015b

**Versions the software has been tested on:**

R2015b

2. Installation guide

**Instructions**: Download the code and open it in matlab.

**Typical install time on a "normal" desktop computer**: a few seconds

3. Demo

**Instructions to run on data:**

Open the image (image1) path in matlab.

Click “*run*”-> choose the desired images-> Choose the desired ROI-> right click->choose “*create mask*”

**Expected output:**

1. A histogram of the eccentricity values


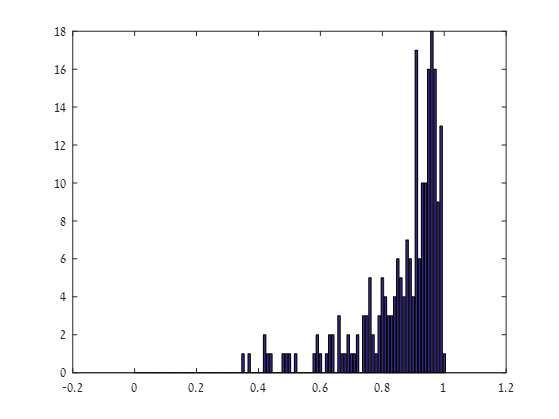


2. An excel file “eccentricity” that includes:

| Image1 | 0.4 | 3.401869 | 0.861542 | 1022427 | 0 | 4.205607 | 12.14953 | 5.607477 | 9.813084 | 12.61682 | 25.23365 | 30.37383 |
| --- | --- | --- | --- | --- | --- | --- | --- | --- | --- | --- | --- | --- |

The last value is the number of elements which contain the eccentricity value that ranges from 0.95-1

**Expected run time for demo on a "normal" desktop computer:** about a minute

4. Instructions for use

**How to run the software on your data:**

- To run on different images, choose the right color channel:

Line 9: im2=double(im(:,:,2)).*ROI/255; (1-for red, 2-for green, 3-for blue)

- Change the threshold value in line 11 according to the quality of your data.
- Open the image path in matlab.
- Click “*run*”-> choose the desired images-> Choose the desired ROI-> right click->choose “*create mask*”
