# Supplementary figures and images for: Genetically engineered human muscle transplant enhances murine host neovascularization and myogenesis
Source: Commun Biol. 2018 Oct 4;1:161. doi: 10.1038/s42003-018-0161-0 (PMC6172230; doi:10.1038/s42003-018-0161-0)

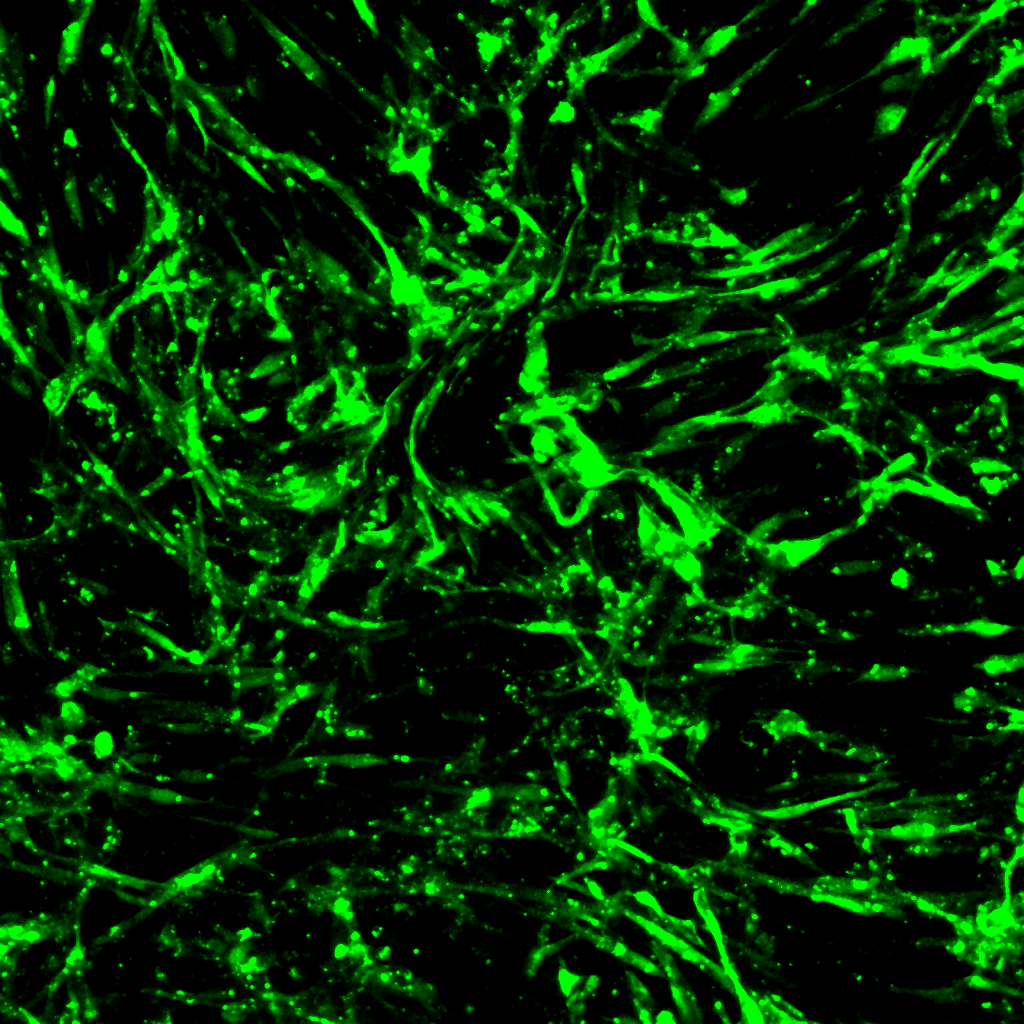

Supplement: Supplementary file 8 — Supplementary Software 2 [file 42003_2018_161_MOESM8_ESM.tif]
